# Comparative Evaluation of Tongue and Periodontal Pocket Microbiome in Relation to *Helicobacter pylori* Gastric Disease: 16S rRNA Gene Sequencing Analysis

**DOI:** 10.3390/antibiotics14080804

**Published:** 2025-08-06

**Authors:** Fausto Zamparini, Alessio Buonavoglia, Francesco Pellegrini, Georgia Diakoudi, Matteo Pavoni, Giulia Fiorini, Vittorio Sambri, Andrea Spinelli, Dino Vaira, Maria Giovanna Gandolfi, Carlo Prati

**Affiliations:** 1 Endodontic Clinical Section, Dental School, Department of Biomedical and Neuromotor Sciences (DIBINEM), University of Bologna, 40125 Bologna, Italy; fausto.zamparini2@unibo.it (F.Z.); alessio.buonavoglia2@unibo.it (A.B.); andrea.spinelli4@unibo.it (A.S.); 2 Laboratory of Green Biomaterials and Oral Pathology, Dental School, Department of Biomedical and Neuromotor Sciences, University of Bologna, 40125 Bologna, Italy; mgiovanna.gandolfi@unibo.it; 3 Department of Veterinary Medicine, University of Bari, 70126 Bari, Italy; francesco.pellegrini@uniba.it (F.P.); georgia.diakoudi@uniba.it (G.D.); 4 Department of Medical and Surgical Sciences, University of Bologna, IRCCS AOUBO, 40138 Bologna, Italy; matteo.pavoni2@unibo.it (M.P.); berardino.vaira@unibo.it (D.V.); 5 Cardiovascular Medicine Unit, IRCCS AOU, 40138 Bologna, Italy; giulia.fiorini@aosp.bo.it; 6 Unit of Microbiology, The Great Romagna Hub Laboratory, 47522 Pievesestina, Italy; vittorio.sambri@unibo.it

**Keywords:** oral bacteria, tongue bacteria, periodontal bacteria, gastric diseases, nanopore sequencing, helicobacter pylori

## Abstract

**Objective:** To analyze the composition of the oral microbiome in periodontal pocket lesions and on the tongue dorsum of patients with *Helicobacter pylori*-associated gastric disease. **Materials and Methods:** Patients diagnosed with gastric disease and *H. pylori* (HP+) were evaluated in comparison to a control group of *H. pylori*-negative patients without gastric disease (HP−). Periodontal and oral health clinical parameters (PPD, BoP, PSE, plaque score and modified DMFT) were assessed for each patient. Microbiological samples were collected from the deepest periodontal pockets and tongue dorsum, followed by DNA extraction, 16S rRNA PCR amplification, and Next-Generation-Sequencing (NGS) analyses. **Results:** Sixty-seven patients (27F; 40M, aged 35–85 years) were enrolled. Of these, 52 were HP+ and 15 were HP−. HP+ patients exhibited a significantly higher presence of decayed teeth (*p* < 0.05) and slightly fewer missing teeth (*p* > 0.05). The plaque score was significantly higher in HP+ patients (*p* < 0.05), while PPD and BoP showed no significant differences (*p* > 0.05). NGS analysis revealed no presence of *H. pylori* in any samples of both periodontal and tongue sites. HP+ patients showed a distinct microbial composition, including higher prevalence of *Capnocytophaga*, *Fusobacterium*, and *Peptostreptococcus* genera in both locations (pockets and tongue dorsum). **Conclusions:** The study demonstrated that HP+ patients exhibit distinct oral microbial profiles compared to HP− patients, especially in areas with deeper periodontal pockets. *H. pylori* was not detected in the oral microbiomes of either group.

## 1. Introduction

Oral microbiota and gastrointestinal microbiota are the most represented microorganism communities of the human body, comprising a large population of bacteria, fungi, protozoa, and viruses that colonize the oral cavity and the gastrointestinal tract and act as commensals or as opportunistic pathogens [1,2,3]. Oral dysbiosis is associated with the overgrowth of some pathogenic bacteria species and with the occurrence of oral pathologies, such as periodontal disease [4,5,6]. The role of a few oral bacterial species in systemic disease has been extensively investigated in relation to cardiovascular disease [7], kidney disease [8], neurodegenerative disease [9,10], or oncological diseases [11].

Periodontal disease is one of the most frequent non-communicable pathologies worldwide [12]. Globally, it is estimated that 20–50% of the world population suffers from periodontal disease [13]. Of these, approximately 10% suffer from severe periodontal disease and are at risk of tooth loss [14]. *Helicobacter pylori* (*H. pylori*) is present in a large percentage of the world population and has been linked to gastric disease and cancer etiopathogenesis [15,16,17]. *H. pylori* prevalence ranges between 85% and 95% in developing countries and between 30% and 50% in developed countries [18,19,20].

It has been hypothesized that gastroesophageal reflux—highly prevalent in the world population [21]—can bring *H. pylori* from the stomach to the oral cavity. The reflux may temporarily bring *H. pylori* or fragments of *H. pylori* from the stomach, leading to positive PCR detection.

Several PCR-based studies revealed the oral colonization in periodontal pockets with *H. pylori*, with a great range of variability (from 0% to 100%) [22,23,24,25,26,27]. However, it is unclear whether *H. pylori* can colonize in the oral cavity. It is also not clear whether *H. pylori* is a permanent resident or simply temporarily present in the oral cavity. The reason for such discrepancies has been recently reported and attributed to the cross-reactivity of *H. pylori*-like microorganisms [such as *Campylobacter* spp., including *Campylobacter cinaedi*, *Campylobacter coli*, *Campylobacter concisus*, *Campylobacter cryaerophila*, *Campylobacter foetus*, *Campylobacter jejuni*, *Campylobacter laridis*, *Campylobacter sputorum subsp. bubulus*, *Campylobacter sputorum subsp. sputorum*, and *Campylobacter upsaliensis*) with the molecular, immunological, or biochemical methods/markers previously used [28]. One study detected *H. pylori* in subgingival plaque samples of 60% of patients with confirmed gastric *H. pylori* infection using PCR but also in 15% of control patients without gastric *H. pylori* infection [29].

Few clinical-microbiological studies are currently present regarding the correlation among oral health, periodontal status, oral microorganisms, and gastric pathologies, as a direct relationship has not been fully validated [3].

The study aimed to analyze the composition of the oral microbiome in patients with different oral health conditions and to assess their positivity to *H. pylori*. This population was compared to healthy patients with no signs of *H. pylori*.

## 2. Results

### 2.1. Periodontal and Oral Health Conditions

A total of 67 patients (27 females, 40 males, aged 35–85 years) were enrolled (132 samples, 65 from the pocket, 67 from the tongue). Of these, 52 were referred to as HP+ and 15 were as HP−.

Table 1 reports the periodontal parameters of patients enrolled in the study. HP+ patients revealed higher percentages of sites with plaque scores (*p* < 0.05), slightly deeper PPD (*p* > 0.05), and slightly higher BoP values (not significantly different from HP−) (*p* > 0.05).

Table 2 reports the examination of the worst periodontal pocket in patients enrolled in the study according to PSE. HP+ patients exhibited higher levels of inflammation, as shown by a greater proportion of PSE scores above 2 and more frequent sites with increased probing pocket depth (PPD) in 67.2% of cases. In contrast, HP− patients more commonly had PSE scores between 1 and 2, accounting for 53.4% of cases.

Table 3 reports the oral health conditions (DMFT) of patients enrolled in the study. HP+ patients had a slightly higher mean number of missing teeth (4.9 ± 5.9) and a significantly greater mean number of decayed teeth (2.3 ± 3.2) compared to HP− patients (*p* = 0.060 and *p* = 0.012, respectively). Conversely, HP− patients exhibited a higher mean number of filled teeth, although this difference was not statistically significant (*p* = 0.441).

### 2.2. Gastric Characteristics of Patients

Table 4 presents the gastric characteristics of the patients included in the study. The HP+ group demonstrated significantly greater progression of gastric disease (*p* = 0.001). In contrast, the HP− group was predominantly characterized by histologically healthy gastric tissue (80%) and a low incidence of esophagitis (20%).

### 2.3. Microbiome Analysis

After quality control of Nanopore sequence data, a total of 58,842,945.0 bacterial 16SrRNA gene sequence reads (mean 502,931.15, median 479,478.0, range 1527.0–1415,529.0) were obtained from the analyzed samples. A total of 60 genera were assigned using Fastq 16S 9 September 2021 workflow, considering only genera above 0.1%.

**Figure 1:**
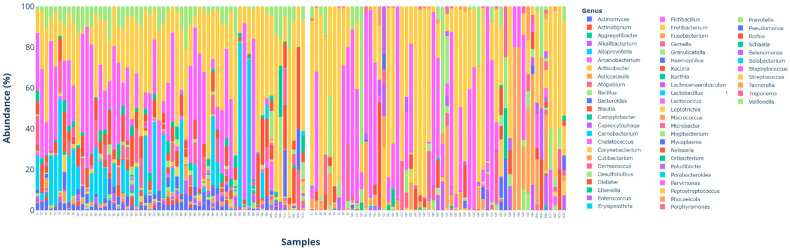
Barplot chart of bacterial genera distribution among tongue (**L**) and pocket (**S**) samples.

The bacterial genera identified in the analyzed samples are reported in Table S1 with distribution expressed as a percentage in different considered categories. Figure 1 presents the Barplot of all collected samples divided between sulcus and tongue.

In none of the oral samples, *H. pylori* DNA was detected.

The most prevalent bacterial genera among the L sample were *Streptococcus*, *Veillonella*, *Actinomyces*, *Rothia*, and *Staphylococcus*, in both HP+ patients and HP− patients.

In S samples, the most prevalent genera in both groups were Streptococcus, Fretibacterium, Selenomonas, Fusobacterium, Parvimonas, Mogibacterium, and Staphylococcus.

In periodontal samples with PPD < 3 mm, the most prevalent bacterial genera detected in both groups were *Streptococcus*, *Parvimonas*, *Selenomonas*, *Fretibacterium*, and *Mogibacterium*.

HP+ patients showed a difference in the presence of *Capnocytophaga*, *Fusobacterium*, and *Peptostreptococcus*, which appeared more prevalent in respect to periodontal samples of HP− patients.

Conversely, periodontal samples of HP− patients showed more prevalence of the bacterial genera *Neisseria* and *Phocaeicola*.

Bacterial genus *Microbacter* (37.50%) was detected only in HP+ patients, while *Dialister* (37.50%), *Porphyromonas* (25%), *Prevotella* (25%), and *Veillonella* (25%) were detected only in HP− patients.

The most prevalent bacterial genera detected in both groups in periodontal pockets with a PPD > 3 mm were *Streptococcus*, *Fretibacterium*, *Staphylococcus*, *Fusobacterium*, and *Selenomonas*. The bacterial genus *Gemella* was detected only in HP− patients (26.32%).

### 2.4. Diversity Indexes

#### 2.4.1. Alpha Diversity

Alpha diversity among the samples, calculated using the Shannon index, ranged between 0.241 and 2.536 (mean, 0.564; median, 0.611), while the biodiversity value using Richness Menhinick’s index ranged between 0.387 and 2.969 (mean, 1.077; median, 0.078). Comparisons of alpha diversity reached the thresholds of statistical significance (*p*  >  0.05) between HP+ and HP− patients with a PPD > 3 mm in the S samples category.

#### 2.4.2. Beta Diversity

**Figure 2:**
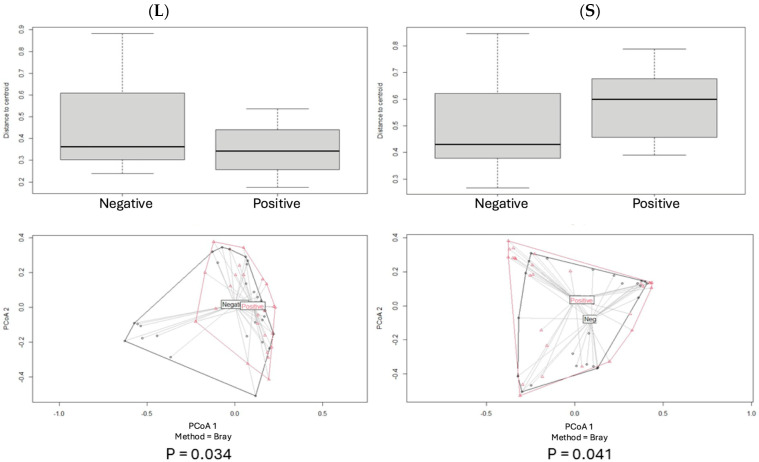
Significant differences were observed between groups in both tongue and pocket samples. Principal Coordinates Analysis (PCoA) of microbial communities on the tongue dorsum (**L**) and in the periodontal sulcus (**S**) of HP+ and HP− patients, based on the Bray–Curtis dissimilarity index. Significant differences in microbial composition were observed between groups for both tongue and sulcus samples.

**Figure 3:**
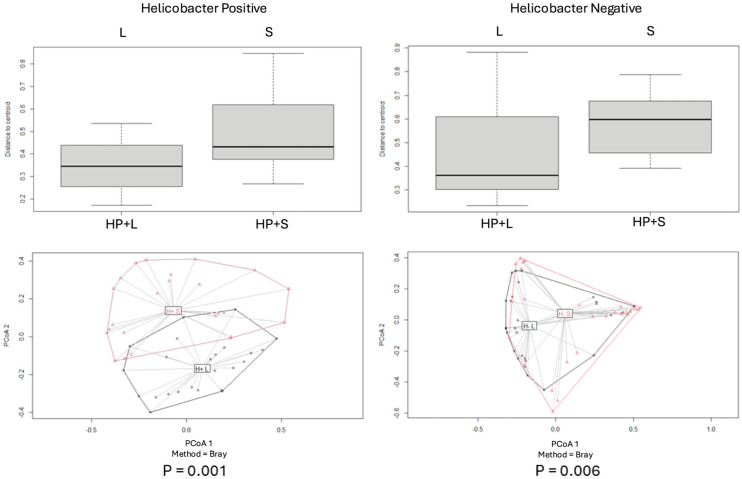
Principal Coordinates Analysis (PCoA) of microbial populations of patients *H. pylori*+ and *H. pylori*− on tongue (**L**) and sulcus (**S**), based on the Bray–Curtis dissimilarity index. Significant differences were observed between the two sites in both HP-positive and -negative groups.

**Figure 4:**
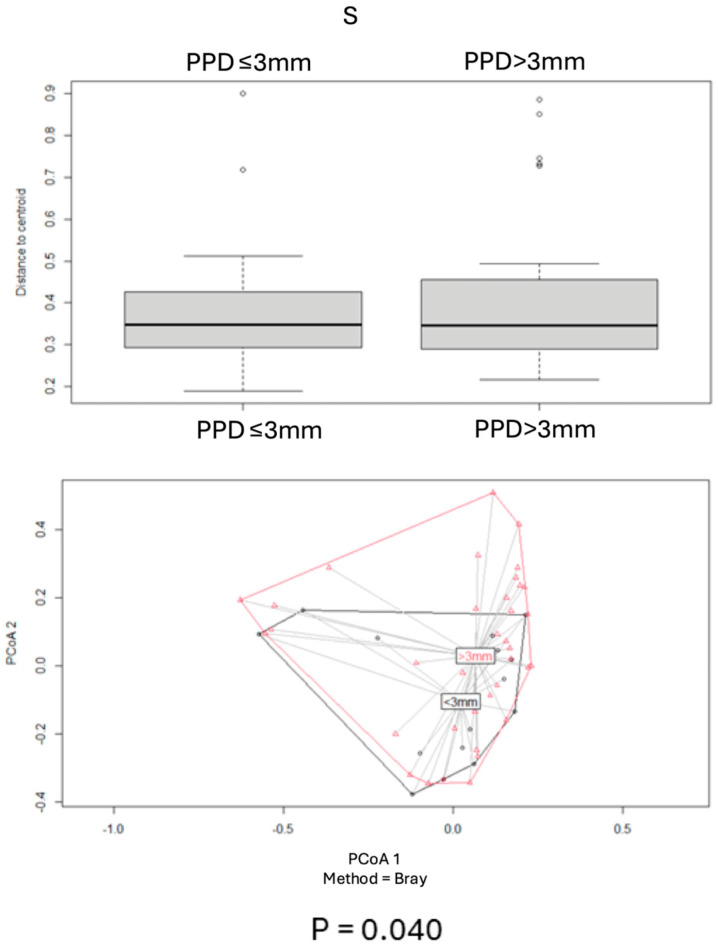
Principal Coordinates Analysis (PCoA) of microbial populations in deep pockets (PPD > 3) and shallow pockets (PPD ≤ 3), based on the Bray–Curtis dissimilarity index. Significant differences were observed between the two groups.

**Figure 5:**
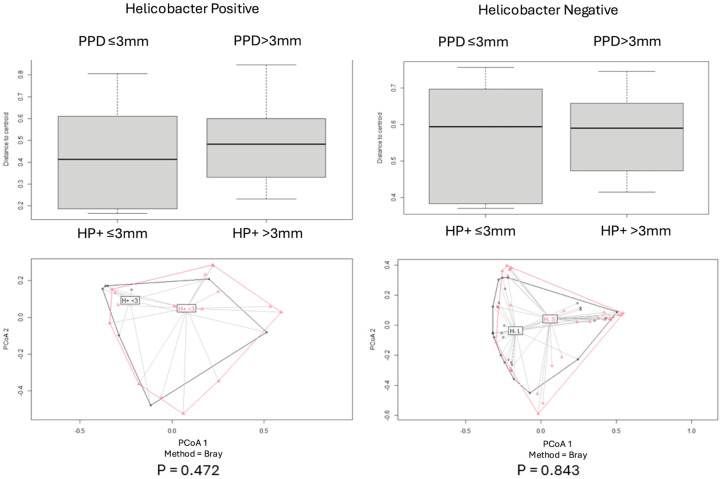
Principal Coordinates Analysis (PCoA) of microbial populations in deep pockets (PPD > 3) and shallow pockets (PPD ≤ 3) of patients with *H. pylori*+ and *H. pylori*, based on the Bray–Curtis dissimilarity index. No significant differences were observed in both groups.

**Figure 6:**
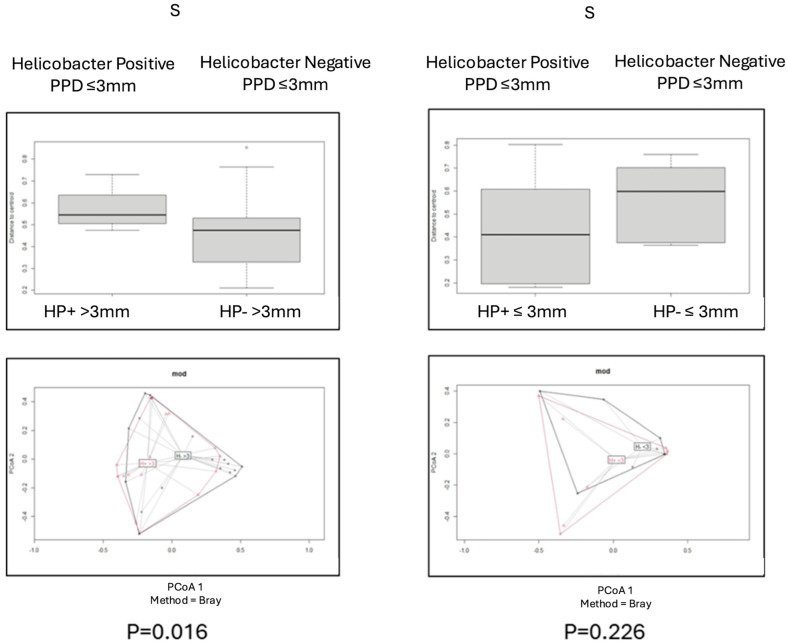
Principal Coordinates Analysis (PCoA) of microbial populations in deep pockets (PPD > 3) of patients *H. pylori*+ and *H. pylori* and shallow pockets (PPD ≤ 3) of patients *H. pylori*+ and *H. pylori*, based on the Bray–Curtis dissimilarity index. Significant differences between HP+ and HP− groups in sites with deep pockets (*p* < 0.05). Non-significant differences were observed between HP+ and HP− groups in sites with shallow pockets.

Beta diversity for each pair of categories was assessed by using the Bray–Curtis index, and PCoA plot graphs were produced (Figure 2, Figure 3, Figure 4, Figure 5 and Figure 6).

Comparisons of beta diversity of samples did not reveal statistical significance (*p* > 0.05) for the categories “PPD ≤ 3 mm vs. PPD > 3 mm” in S samples (Figure 4), “PPD ≤ 3 mm vs. PPD > 3 mm” in HP+ samples, “PPD ≤ 3 mm vs. PPD > 3 mm” in HP− samples (Figure 5), and “HP+ vs. HP−“ in S samples with a PPD ≤ 3 mm (Figure 6).

Comparisons of beta diversity of samples highlighted statistical significance (*p* < 0.05) for the categories “HP+ vs. HP−” in S samples, “HP+ vs. HP−” in L samples (Figure 2), “HP+ L vs. S; HP− L vs. S” samples (Figure 3), and “HP+ vs. HP−” in S samples with a PPD > 3 mm (Figure 6). To further validate these findings, the *p*-values were adjusted using the Benjamini–Hochberg method to control for the False Discovery Rate (FDR), confirming statistical significance for the comparison between “HP+ and HP−” in S samples (q = 0.0083), “HP+ and HP−” in L samples (q = 0.0269), and for the comparison between “HP+ and HP−” in S samples with a PPD > 3 mm (q = 0.0471).

## 3. Discussion

NGS has improved the knowledge on the microbiota thriving in several districts of the human body in either physiological or pathological conditions [30]. In the present study, the analysis of the oral microbiome was performed using NGS to investigate the microbiome composition in samples obtained from the tongue dorsum and periodontal pocket using 16S rDNA analysis on the MinION platform of ONT.

The rationale for the study was based on the hypothesis that gastric infection of *H. pylori* could be related to a concomitant presence in oral cavity of this bacterium. Moreover, oral microbiome composition and periodontal conditions may be different in patients affected by HP+ persistent gastric disease versus HP− patients.

The study therefore described the oral health conditions and the microbiome of HP+ patients in comparison to patients with no gastric pathology and no *H. pylori* colonization (HP−). The tongue dorsum and the gingival sulcus/pocket were separately analyzed as *loci/niches* that could potentially harbor different gram-negative and anaerobic bacteria. The deepest portions of the tongue were rarely investigated in the literature, and no studies analyzed the biofilm of *H. pylori*-affected patients. The samples from the tongue were obtained through a “hard” scraping in order to collect both the superficial and the deepest biofilm. The tongue may act as a reservoir of bacteria that might be ingested and disseminated in the esophagus and the gut [31]. Samples from the periodontal pockets were retrieved using sterile paper cones. Analysis of “shallow” (PPD less or equal to 3 mm) or deep (PPD more than 3 mm) periodontal pockets microbiomes has been recently investigated [32]. The 3 mm cut off, representative of a different microbial community due to a greater presence of inflammation and proliferation of anaerobic bacteria, has been applied and implemented with the analysis of bleeding sites (where inflammation is active) and plaque score (where a more consistent biofilm is revealed).

In this study *H. pylori* was not detected in any oral sample of tongue dorsum and periodontal pocket. The inspection of oral microbiota of the tongue was rare in clinical studies. On the contrary, the evaluation of *H. pylori* in periodontal pockets was hypothesized and observed by previous investigations. PCR-based studies revealed *H. pylori* presence in periodontal pockets [22,23,24,25,26,27,33]. Cross-reactivity of the markers to *Helicobacter*-like microorganisms (such as *Campylobacter*) could partially explain these results [28]. Alpha and beta diversity analyses were applied to explore possible trends in the 16S rDNA data generated from the patients enrolled in this study. In these analyses, there was a significant difference in the oral microbiome in terms of changes in composition, both on the tongue and in periodontal pockets with a depth > 3 mm.

Our study identified a distinctly different microbiome on the tongue dorsum when compared to the periodontal pocket, characterized by a high relative abundance of the genera *Streptococcus*, *Veillonella*, *Actinomyces*, *Rothia*, and *Staphylococcus*. These taxa were consistently prevalent in tongue samples from both HP+ and HP− patients.

In contrast, the subgingival microbiome in HP+ patients—particularly in deep periodontal pockets—differed significantly from that of HP− patients. Notably, *Dialister* (37.5%), *Porphyromonas* (25%), *Prevotella* (25%), and *Veillonella* (25%) were detected exclusively in HP− deep pocket samples. Overall, HP+ patients with pocket depths greater than 3 mm (PPD > 3 mm) exhibited a significantly altered microbial profile compared to HP− individuals (Figure 6).

These results can be explained by the markedly higher presence of plaque and mineralized tissue in HP+ patients, detected by plaque score, and agree with a recent microbiological study on deep and shallow pockets [32]. Therefore, this study confirms that gingival sulcus/pocket had a markedly different composition when sites with PPD greater than 3 mm are considered.

The pH range of the gingival sulcus has been reported to vary between 6.5 and 8.5, and increased pocket depth and inflammation have been correlated with increased alkalinization. In the deepest pockets, the pH rises to 8.9 [34,35]. Only limited bacterial species can colonize this site, such as *Fusobacterium nucleatum* and Red Complex bacteria. Interestingly, the deep periodontal pockets of HP+ patients showed a higher abundance of *Capnocytophaga*, *Fusobacterium*, and *Peptostreptococcus* genera, whereas Red Complex-associated genera, such as *Porphyromonas* and *Prevotella* were scarcely detected. Among the enriched taxa, *Fusobacterium* was predominant and is known as a mid-to-late colonizer of the periodontal pocket. Notably, *Fusobacterium nucleatum* displays strong acid-neutralizing activity [36], and while it typically exists in a planktonic state at pH levels below 8, it tends to form aggregates at pH ~8.2, facilitating colonization by other anaerobic Gram-negative bacteria in deep subgingival environments [37]. *Fusobacterium* has also been implicated in gastric inflammation and colorectal cancer, with overrepresentation reported in both fecal samples and tumor tissues [37,38].

*Capnocytophaga* showed higher relative abundance than *Fusobacterium* in shallow pockets (PPD < 3 mm) of HP+ patients. Although its systemic involvement is limited, *Capnocytophaga* plays a role in periodontal infections in immunocompetent individuals and is frequently isolated from periodontal pockets and abscesses, often alongside other pathogenic species [39]. This condition contributes to alveolar bone loss, attachment loss, tooth mobility, and eventual tooth loss. *Peptostreptococcus*, while a common commensal organism, has been associated with chronic infections, and *P. micros* in particular shows a moderate link to periodontal disease [40,41,42,43].

In HP− patients, the deepest periodontal pockets were more commonly associated with *Prevotella*, *Treponema*, and *Porphyromonas*—all well-known periodontitis-associated genera. *Prevotella* species have been implicated in a variety of infections, including aspiration pneumonia, pulmonary abscesses, chronic otitis media, and sinusitis [44]. *P. denticola* has been strongly linked to severe periodontal and endodontic infections [45,46], and its presence has also been associated with gut inflammation and dysbiosis [47,48]. One study reported that a *Prevotella*-dominant gut microbiota increased host susceptibility to mucosal inflammation [49].

*Treponema* species are frequently encountered in the oral cavity and associated with periodontitis. This genus colonizes the gastrointestinal tract [34]. Notably, its presence in the gut microbiome of Western populations has been seldom reported [50].

*Porphyromonas gingivalis*, a recurring species in deep periodontal pockets, has also been detected in other chronic oral pathologies, such as non-healing periapical lesions [46]. Beyond the oral cavity, it has been isolated from the gastrointestinal tract [51], genitourinary tract [52], and even brain biopsies of patients with Alzheimer’s disease [10].

Overall, these microbiological findings warrant consideration, especially given that HP+ patients exhibited poorer oral health conditions, as evidenced by both DMFI and periodontal parameters. Indeed, the reasons for such different microbiome compositions could be directly influenced by patient oral health conditions. In our study HP+ patients demonstrated a significantly higher presence of dental decays, a greater (but not significant) presence of missed teeth and a lower presence of filled teeth when compared to HP− patients, a population with the same age and characteristics. These data could indicate a higher susceptibility of these patients to develop dental caries and higher plaque accumulation when compared to healthy patients. It should be specified that all included patients did not report antibiotic assumption in the month before oral examination and had a similar dental recall rate (no dental hygiene performed in the last 6–9 months).

A possible interpretation of these data is that *H. pylori* gastric infection could determine a shift of composition in the oral microbiome. *H. pylori* infection may be able to interfere with oral microbiome homeostasis by modifying the normal microflora of the stomach and through the interaction that *H. pylori* (and its supernatants) may have with oral bacteria [53,54]. Recurrent gastroesophageal reflux could induce higher prevalence of dental caries due to a more acidic oral health environment, decreased salivation, and disruption of its buffering capacity [55].

From a future research perspective, oral microbiome analyses should focus on comparing patients with different stages of gastric disease, particularly those with precancerous gastric lesions. Larger studies with balanced case–control ratios are needed to validate the current findings and enhance statistical power. Additionally, the poorer oral health conditions observed in patients with gastric disease highlight the need to prioritize this population in structured follow-up and preventive care protocols.

## 4. Materials and Methods

### 4.1. Study Design and Sample

The study had a case-control observational design and was conducted in two university departments from May 2021 to December 2022. The research was carried out by the same clinical staff throughout the study period. The study was approved by the local ethical committee (845-2021-OSS-AUSLBO-21161-ID 3117 Acronym: ORAL-GASTOBIOTA).

All patients included in this investigation were treated according to the principles established by the Declaration of Helsinki as modified in 2013 [56]. Before enrollment, written and verbal information was given by the clinical staff, and each patient gave written consent according to the above-mentioned principles.

This report was written according to Strengthening the Reporting of Observational Studies in Epidemiology guidelines [57].

### 4.2. Patient Selection

Volunteer patients from the Department of Medical and Surgical Sciences (DIMEC), University of Bologna, were recruited and screened for the presence of *H. pylori* and gastric pathologies through a breath test and a gastric biopsy. Patients were then enrolled if they fulfilled the following inclusion and exclusion criteria:

Inclusion criteria:

- Age between 35 and 85 years
- Breath test performed within 1 week
- Gastric biopsy
- ASA status 1–2
- Local geographic provenience

Exclusion criteria:

- Age below 35 years
- Negative breath test performed within 1 week+
- Absence of gastric pathologies
- Edentulous patients

### 4.3. Clinical Parameters

Patients were then referred to the Endodontic Clinical Section—University Dental School, Department of Biomedical and Neurological Sciences (DIBINEM), University of Bologna. A full clinical examination of the oral cavity was performed for each patient. Anamnestic data were obtained and recorded in the patient’s clinical chart. Information regarding the dentition status and periodontal status was also recorded.

#### 4.3.1. Dentition Status

The Decayed, Missing, and Filled Teeth (DMFT) index has been used to describe the current dentition status of the enrolled patients. DMFT provides the sum of decayed, missing, and filled permanent teeth of an individual [58]. DMFT was slightly adapted, including the evaluation of the presence of implants (Decayed, Missing, Filled Teeth, and Implants, DMFTI).

#### 4.3.2. Periodontal Status

The periodontal status has been defined by pocket probing depth (PPD), plaque index (PI), and bleeding on probing (BoP).

- *Plaque score (plaque score):* The presence of plaque or calculus was assessed at mesial, distal, vestibular and palatal sites around each tooth. The score was 0 = no plaque; 1 = thin layer of plaque; 2 = moderate layer of plaque and calculus; 3 = abundant plaque and calculus) [59].
- *Bleeding on probing (BoP):* The presence of bleeding on probing was measured at mesial, distal, vestibular, and palatal sites around each tooth. The scores were (0 = no bleeding; 1 = bleeding) [60]. The total percentage of sites with BoP = 1 was then recorded and grouped in quartiles: Q1 = 0–25%, Q2 = 26–50%, Q3 = 51–75%, and Q4 = 76–100% of sites.
- *Pocket probing depth (PPD):* The measurements were made using a periodontal probe with a light force (approximately 0.25 N) in mesial distal vestibular and palatal sites [61]. The deepest PPD was recorded in mm.

A cumulative periodontal status (periodontal screening and record, PSE) was then calculated as follows [62]:

(1) No pockets exceeding 3 mm (PP < 3 mm), no calculus (plaque score = 0), but presence of bleeding on probing (BoP = 1).
(2) No pockets exceeding 3 mm (PPD < 3 mm), but calculus or other plaque-retentive factors detected (PI = 1–3), with or without bleeding on probing (BoP = 0–1).
(3) Pockets comprised within 3.5 mm and 5.5 mm (PPD= 3.5–5.5 mm) with or without plaque, calculus, or bleeding on probing.
(4) Pocket comprised within 5.5 mm and 7 mm in depth (PPD = 5.5–7 mm) with or without plaque, calculus or bleeding on probing.
(5) Pockets over 7 mm (PPD > 7 mm) and/or furcation involvement with or without plaque, calculus, or bleeding on probing.

PSE was used in order to select the site with the worst periodontal conditions in terms of pocket depth and inflammation.

Sites categorized as PSE 1–2 were defined as healthy, while sites with a PSE 3–5 were categorized as periodontally affected.

### 4.4. Samples Collection and Microbiome Analysis

Sterile paper points (#25) were used for the microbial sample collection at the pocket and tongue sites.

For the pocket sites (S), the paper point was inserted in the deepest portion of the selected pocket and maintained in situ for approximately 30 s.

For the tongue sites (L), a sterile tongue scraper was used at the middle third of the dorsal portion of the tongue. The sterile paper points were used to collect the microbiological samples from the tongue scraper.

The paper points samples were then inserted in sterile tubes (Eppendorf, AG, Hamburg, Germany) and subsequently stored at −20 °C until use.

Microbiological analysis evaluated oral bacterial composition of *H pylori*+ patients (HP+) and *H. pylori*− patients (HP−) on the dorsum of the tongue (L) and in periodontal pocket (S). Moreover, periodontal samples were divided into two subgroups depending on periodontal probing depth (PPD).

### 4.5. Genome Extraction

The Eppendorf tubes were filled with Dulbecco’s Minimal Essential Medium (DMEM). These samples were then blended using a Tissue Lyser (Qiagen GmbH, Hilden, Germany) at 30 Hz for 5 minutes. The homogenized samples underwent centrifugation at 10,000× *g* for 3 min. Following this, 200 µL of the cleared liquid (supernatant) were used for DNA extraction with the Dneasy PowerSoil PRO kit (Qiagen S.p.A., Milan, Italy), following the provided instructions. Negative controls consisting of sterile saline solution were processed alongside the clinical samples using the same protocol.

### 4.6. PCR Amplification of the 16SrDNA Gene and Nanopore Sequencing

DNA extracts underwent a PCR protocol to amplify the complete (1500 bp) sequence of the 16S rRNA gene employing universal primers 27F and 1492R and the TaKaRa LA TaqTM kit from Takara Bio Europe S.A.S., Saint-Germain-en-Laye, France. Length distributions of the obtained amplicons were evaluated by an A2100 Bioanalyzer (Agilent Technologies, Santa Clara, CA, USA) with a High Sensitivity DNA chip, while DNA concentration was evaluated in a Qubit 4.0 Fluorometer using a Qubit dsDNA HS kit (Invitrogen, Life Technologies, Milan, Italy), both according to the manufacturer’s instructions.

Subsequently, libraries were prepared using the 16S barcoding kit SQK-RAB204 from Oxford Nanopore Technologies (ONT, Oxford, UK). These libraries were purified using Agencourt AMPure XP magnetic beads from Beckman Coulter™, combined, and then sequenced using a MinION flongle Flow cell FLO-FLG001, version R9.4.1 adapted for the MinION- Mk1C device (ONT, Oxford, UK) for a duration of 24 h.

### 4.7. Data Analysis and Visualization

FastQ MinION files were uploaded on the online EPI2ME platform (https://epi2me.nanoporetech.com/ (accessed on 5 May 2024)) and analyzed by the Fastq 16S 2021.09.09 (Metrichor Agent, ONT) workflow setting the following parameters: quality score 10, minimum length filter of 1500 bases, and BLAST E-value of 0.01.

The taxonomy was assigned by using the NCBI 16S database (ncbi_16s_18s) through the BLAST tool (ver. 2.5.6) with a minimum horizontal coverage of 30% and a minimum accuracy of 77% as default parameters. The data obtained were organized in Microsoft Office Excel sheets, considering only taxa with a ≥0.1% relative abundance in samples. Further analyses were carried out by using the “Plotly.py” open-source library for Python 3.8.1 [63]. The relative % data were represented as interrogable BarPlot charts and Pie Chart. Statistical analyses were conducted using R version 4.1.3 and the “vegan” library (https://vegandevs.github.io/vegan/ (accessed on 5 May 2024)).

#### 4.7.1. Alpha Diversity

To assess the community’s composition, alpha diversity was determined for each sample through the evaluation of the Shannon index, while the measurement of biodiversity utilized the Richness Menhinick’s index. The normality of data distribution was assessed using the Shapiro–Wilk test. A two-sided Student’s *t*-test for independent samples or a Mann–Whitney U test was employed to analyze alpha diversity and biodiversity values based on predefined categories.

#### 4.7.2. Beta Diversity

For potential sample stratification, beta diversity was evaluated using the Bray–Curtis index. Principal Coordinate Analysis (PCoA) was then used to visualize the stratification of the samples. To test for statistically significant differences in microbial composition among the predefined categories, a permutational multivariate analysis of variance (PERMANOVA) was performed using the adonis function within the ‘vegan’ package in R, with 999 permutations. The *p*-values obtained from the PERMANOVA tests were subsequently adjusted for multiple comparisons using the Benjamini–Hochberg method to control the false discovery rate (FDR). A corrected *p*-value (q-value) of less than 0.05 was considered statistically significant.

#### 4.7.3. Statistical Analysis

Clinical data were recorded in an Excel file and analyzed using Stata 17.1 (StataCorp, College Station, TX, USA). Data reported as values and percentages (BoP, plaque score, PSE, Perio, histology, and presence of *H. pylori*) were analyzed through the Chi-Square test (*p* < 0.05). Data reported as mean ± standard deviations (PPD, DMFT values) were analyzed through one-way ANOVA with the Student–Newman–Keuls post hoc test (*p* < 0.05).

## 5. Conclusions

The study identified two distinct microbiota profiles and bacterial populations between the patient groups (HP+ versus HP−), as evidenced by beta and alpha diversity indices.

In HP+ patients, the genera *Capnocytophaga*, *Fusobacterium*, and *Peptostreptococcus* were more prevalent compared to the periodontal samples of HP− patients. Interestingly, *Porphyromonas* and *Prevotella* were present only in limited amounts in the HP+ group.

No *H. pylori* DNA was detected in either the tongue or the periodontal pockets, suggesting these oral sites are unlikely to serve as reservoirs for the bacterium.

The tongue exhibited high variability and contained only a limited range of specific periodontal bacteria.

## Figures and Tables

**Table 1:** Periodontal parameters (BoP, plaque scores, expressed as *n* and %), and pocket probing depth (PPD, expressed as mean ± SD).

		HP+ *n* = 52		HP− *n* = 15		Total *n* = 67		*p*-Value **
	Values	*n*	%	*n*	%	*n*	%	
BoP	0–25	25	48.1	9	60.0	32	47.7	0.739
	26–50	16	30.7	5	33.3	23	34.3	
	51–75	7	13.5	1	6.6	8	11.9	
	>75	2	3.8	0	0	2	2.9	
	NA *	2	3.8	0	0	4	5.8	
Plaque score	0	4	7.6	0	0	4	5.8	**0.005**
	1	9	17.3	3	20.0	12	17.9	
	2	11	21.1	10	66.6	19	28.3	
	3	27	51.9	2	13.3	29	43.2	
	NA *	2	3.8	0	0	4	5.8	
PPD (Mean ± SD)		5.58 ± 2.9		5.53 ± 2.8		5.49 ± 2.9		0.95

* Totally edentulous; ** Chi square test was performed for BoP and plaque score, and ANOVA was performed for PPD (*p* < 0.05).

**Table 2:** PSE and periodontal pocket depth (shallow/deep) (expressed as *n* and %).

		HP+ *n* = 52		HP− *n* = 15		Total *n* = 67		*p*-Value **
	Values	*n*	%	*n*	%	*n*	%	
PSE	1	2	3.8	3	20.0	5	7.5	0.346
	2	12	23.1	4	33.3	16	23.8	
	3	13	25.0	3	26.6	16	23.8	
	4	11	21.1	1	6.6	12	17.9	
	5	12	23.1	4	26.6	16	23.9	
	NA *	2	3.8	0	0	2	2.9	
Shallow Deep	≤3 mm	14	26.9	7	46.6	22	32.8	0.177
	>3 mm	36	69.2	8	53.4	45	67.2	
	NA *	2	3.9					

* Totally edentulous; ** Chi-square test (*p* < 0.05).

**Table 3:** Oral health conditions (DMFT expressed as mean ± SD) of patients.

	HP+ *n* = 52	HP− *n* = 15	Total *n* = 67	*p*-Value **
Decayed (D)	2.3 ± 3.2	1.2 ± 3.8	2.1 ± 3.1	**0.012**
Missing (M)	4.9 ± 4.9	2 ± 4.9	4.2 ± 5.9	0.060
Filled (F)	4.2 ± 4.9	6.93 ± 5.9	4.8 ± 4.9	0.058
Implant (I)	1.09 ± 2.1	0.93 ± 2.5	1.05 ± 2.1	0.800
DMFI	12.6 ± 6.8	11.5 ± 6.6	12.1 ± 7.9	0.441

** ANOVA test (*p* < 0.05).

**Table 4:** Gastric characteristics (expressed as *n* and %) of patients.

		HP+ *n* = 52		HP− *n* = 15		Total *n* = 67		*p*-Value **
	Values	*n*	%	*n*	%	*n*	%	
Histology	Esophagitis	1	1.9	3	20	4	5.9	**0.001**
	Healthy	0	0	0	0	0	0	
	Quiescent	3	5.7	12	80	15	22.3	
	Mild *	23	44.2	0	0	23	34.3	
	Moderate	16	30.7	0	0	16	23.8	
	Severe	9	17.3	0	0	9	19.3	
	Metaplasia	0	0	0	0	0	0	
HP	-	1	19.2	15	100	16	23.8	**0.001**
	+	4	7.6	0	0	4	5.9	
	++	25	48.1	0	0	25	37.3	
	+++	22	42.3	0	0	22	32.7	

* Totally edentulous; ** Chi-square test (*p* < 0.05).

## Data Availability

Data available upon reasonable request.

## Supplementary Materials

The following supporting information can be downloaded at: https://www.mdpi.com/article/10.3390/antibiotics14080804/s1, Table S1: Bacterial Genera Identified in the Analyzed Samples and Their Relative Abundance.
