# Supplementary material for: Comparative Evaluation of Tongue and Periodontal Pocket Microbiome in Relation to Helicobacter pylori Gastric Disease: 16S rRNA Gene Sequencing Analysis
Source: Antibiotics (Basel). 2025 Aug 6;14(8):804. doi: 10.3390/antibiotics14080804 (PMC12382620; doi:10.3390/antibiotics14080804)
Supplement: Supplementary file 1 [file antibiotics-14-00804-s001.zip › antibiotics-3753118-supplementary.pdf]

Comparative Evaluation of Tongue and Periodontal Pocket Microbiome in Relation to Helicobacter pylori Gastric Disease: 16S rRNA Gene Sequencing Analysis

Supplementary Materials

Table S1 Bacterial Genera Identified in the Analyzed Samples and Their Relative Abundance

Type	Total		L		S		S		Helicobacter +		Helicobacter -		Helicobacter +		Helicobacter -	
	L	S	Helicobacter +	Helicobacter -	Helicobacter +	Helicobacter -	PP ≤ 3	PP > 3	L	S	L	S	PP ≤ 3	PP > 3	PP ≤ 3	PP > 3
Actinomyces	86.54%	5.88%	100.00%	75.00%	4.17%	3.85%	11.76%	2.94%	100.00%	4.17%	75.00%	7.41%	12.50%	0.00%	12.50%	5.26%
Actinotignum	23.08%	0.00%	16.67%	28.57%	0.00%	0.00%	0.00%	0.00%	16.67%	0.00%	28.57%	0.00%	0.00%	0.00%	0.00%	0.00%
Aggregatibacter	3.85%	0.00%	4.17%	3.57%	0.00%	0.00%	0.00%	0.00%	4.17%	0.00%	3.57%	0.00%	0.00%	0.00%	0.00%	0.00%
Alkalibacterium	5.77%	3.92%	4.17%	7.14%	8.33%	0.00%	5.88%	2.94%	4.17%	8.33%	7.14%	0.00%	12.50%	6.25%	0.00%	0.00%
Alloprevotella	3.85%	5.88%	4.17%	3.57%	8.33%	3.85%	0.00%	8.82%	4.17%	8.33%	3.57%	3.70%	0.00%	12.50%	0.00%	5.26%
Arcanobacterium	11.54%	3.92%	8.33%	14.29%	4.17%	3.85%	0.00%	5.88%	8.33%	4.17%	14.29%	3.70%	0.00%	6.25%	0.00%	5.26%
Arthrobacter	3.85%	3.92%	4.17%	3.57%	8.33%	0.00%	0.00%	5.88%	4.17%	8.33%	3.57%	0.00%	0.00%	12.50%	0.00%	0.00%
Asticcacaulis	0.00%	3.92%	0.00%	0.00%	4.17%	3.85%	0.00%	5.88%	0.00%	4.17%	0.00%	3.70%	0.00%	6.25%	0.00%	5.26%
Atopobium	28.85%	1.96%	20.83%	35.71%	0.00%	3.85%	0.00%	2.94%	20.83%	0.00%	35.71%	3.70%	0.00%	0.00%	0.00%	5.26%
Bacillus	38.46%	3.92%	41.67%	35.71%	4.17%	3.85%	5.88%	2.94%	41.67%	4.17%	35.71%	3.70%	12.50%	0.00%	0.00%	5.26%
Bacteroides	1.92%	3.92%	4.17%	0.00%	4.17%	3.85%	5.88%	2.94%	4.17%	4.17%	0.00%	3.70%	12.50%	0.00%	0.00%	5.26%
Blautia	11.54%	0.00%	12.50%	10.71%	0.00%	0.00%	0.00%	0.00%	12.50%	0.00%	10.71%	0.00%	0.00%	0.00%	0.00%	0.00%
Campylobacter	11.54%	9.80%	16.67%	7.14%	8.33%	11.54%	5.88%	11.76%	16.67%	8.33%	7.14%	11.11%	12.50%	6.25%	0.00%	15.79%
Capnocytophaga	13.46%	15.69%	12.50%	14.29%	16.67%	11.54%	35.29%	5.88%	12.50%	16.67%	14.29%	14.81%	50.00%	0.00%	25.00%	10.53%
Carnobacterium	9.62%	0.00%	12.50%	7.14%	0.00%	0.00%	0.00%	0.00%	12.50%	0.00%	7.14%	0.00%	0.00%	0.00%	0.00%	0.00%
Chelatococcus	3.85%	0.00%	0.00%	7.14%	0.00%	0.00%	0.00%	0.00%	0.00%	0.00%	7.14%	0.00%	0.00%	0.00%	0.00%	0.00%
Corynebacterium	3.85%	7.84%	0.00%	7.14%	4.17%	7.69%	5.88%	8.82%	0.00%	4.17%	7.14%	11.11%	0.00%	6.25%	12.50%	10.53%
Cutibacterium	9.62%	0.00%	16.67%	3.57%	0.00%	0.00%	0.00%	0.00%	16.67%	0.00%	3.57%	0.00%	0.00%	0.00%	0.00%	0.00%
Dermacoccus	11.54%	0.00%	16.67%	7.14%	0.00%	0.00%	0.00%	0.00%	16.67%	0.00%	7.14%	0.00%	0.00%	0.00%	0.00%	0.00%
Desulfobulbus	1.92%	7.84%	0.00%	3.57%	8.33%	7.69%	0.00%	11.76%	0.00%	8.33%	3.57%	7.41%	0.00%	12.50%	0.00%	10.53%
Desulfomicrobiu	1.92%	0.00%	0.00%	3.57%	0.00%	0.00%	0.00%	0.00%	0.00%	0.00%	3.57%	0.00%	0.00%	0.00%	0.00%	0.00%
Dialister	0.00%	17.65%	0.00%	0.00%	16.67%	19.23%	17.65%	17.65%	0.00%	16.67%	0.00%	18.52%	0.00%	25.00%	37.50%	10.53%
Eikenella	5.77%	5.88%	0.00%	10.71%	4.17%	7.69%	5.88%	5.88%	0.00%	4.17%	10.71%	7.41%	12.50%	0.00%	0.00%	10.53%
Enterococcus	38.46%	1.96%	29.17%	46.43%	0.00%	3.85%	0.00%	2.94%	29.17%	0.00%	46.43%	3.70%	0.00%	0.00%	0.00%	5.26%
Erysipelothrix	9.62%	0.00%	8.33%	10.71%	0.00%	0.00%	0.00%	0.00%	8.33%	0.00%	10.71%	0.00%	0.00%	0.00%	0.00%	0.00%
Fictibacillus	7.69%	5.88%	4.17%	10.71%	4.17%	7.69%	0.00%	8.82%	4.17%	4.17%	10.71%	7.41%	0.00%	6.25%	0.00%	10.53%
Fretibacterium	55.77%	56.86%	75.00%	39.29%	54.17%	57.69%	64.71%	52.94%	75.00%	54.17%	39.29%	59.26%	50.00%	56.25%	75.00%	52.63%
Fusobacterium	51.92%	47.06%	50.00%	53.57%	50.00%	42.31%	29.41%	55.88%	50.00%	50.00%	53.57%	44.44%	50.00%	50.00%	12.50%	57.89%
Gemella	57.69%	11.76%	62.50%	57.14%	0.00%	23.08%	5.88%	14.71%	62.50%	0.00%	57.14%	22.22%	0.00%	0.00%	12.50%	26.32%
Granulicatella	42.31%	15.69%	41.67%	42.86%	8.33%	23.08%	23.53%	11.76%	41.67%	8.33%	42.86%	22.22%	12.50%	6.25%	25.00%	21.05%
Haemophilus	55.77%	7.84%	58.33%	57.14%	12.50%	3.85%	5.88%	8.82%	58.33%	12.50%	57.14%	3.70%	12.50%	12.50%	0.00%	5.26%
Kocuria	21.15%	0.00%	20.83%	21.43%	0.00%	0.00%	0.00%	0.00%	20.83%	0.00%	21.43%	0.00%	0.00%	0.00%	0.00%	0.00%
Kurthia	13.46%	0.00%	12.50%	14.29%	0.00%	0.00%	0.00%	0.00%	12.50%	0.00%	14.29%	0.00%	0.00%	0.00%	0.00%	0.00%
Lachnoanaerobac	15.38%	9.80%	20.83%	10.71%	12.50%	7.69%	17.65%	5.88%	20.83%	12.50%	10.71%	7.41%	25.00%	6.25%	12.50%	5.26%
Lactobacillus	57.69%	0.00%	62.50%	53.57%	0.00%	0.00%	0.00%	0.00%	62.50%	0.00%	53.57%	0.00%	0.00%	0.00%	0.00%	0.00%
Lactococcus	30.77%	9.80%	25.00%	35.71%	12.50%	3.85%	23.53%	2.94%	25.00%	12.50%	35.71%	7.41%	37.50%	0.00%	12.50%	5.26%
Leptotrichia	38.46%	7.84%	37.50%	39.29%	8.33%	7.69%	0.00%	11.76%	37.50%	8.33%	39.29%	7.41%	0.00%	12.50%	0.00%	10.53%
Macrococcus	7.69%	0.00%	8.33%	7.14%	0.00%	0.00%	0.00%	0.00%	8.33%	0.00%	7.14%	0.00%	0.00%	0.00%	0.00%	0.00%
Microbacter	0.00%	15.69%	0.00%	0.00%	25.00%	7.69%	17.65%	14.71%	0.00%	25.00%	0.00%	7.41%	37.50%	18.75%	0.00%	10.53%
Mogibacterium	46.15%	43.14%	45.83%	46.43%	45.83%	38.46%	47.06%	41.18%	45.83%	45.83%	46.43%	40.74%	50.00%	43.75%	50.00%	36.84%
Mycoplasma	15.38%	17.65%	16.67%	14.29%	16.67%	19.23%	17.65%	17.65%	16.67%	16.67%	14.29%	18.52%	12.50%	18.75%	12.50%	21.05%
Neisseria	40.38%	35.29%	29.17%	50.00%	33.33%	38.46%	41.18%	32.35%	29.17%	33.33%	50.00%	37.04%	37.50%	31.25%	50.00%	31.58%
Oribacterium	38.46%	0.00%	41.67%	35.71%	0.00%	0.00%	0.00%	0.00%	41.67%	0.00%	35.71%	0.00%	0.00%	0.00%	0.00%	0.00%
Paludibacter	0.00%	21.57%	0.00%	0.00%	25.00%	19.23%	5.88%	29.41%	0.00%	25.00%	0.00%	18.52%	0.00%	37.50%	12.50%	21.05%
Parabacteroides	15.38%	0.00%	12.50%	17.86%	0.00%	0.00%	0.00%	0.00%	12.50%	0.00%	17.86%	0.00%	0.00%	0.00%	0.00%	0.00%
Parvimonas	30.77%	47.06%	25.00%	35.71%	50.00%	42.31%	58.82%	41.18%	25.00%	50.00%	35.71%	44.44%	75.00%	37.50%	50.00%	42.11%
Peptostreptococ	30.77%	19.61%	29.17%	32.14%	25.00%	15.38%	29.41%	14.71%	29.17%	25.00%	32.14%	14.81%	50.00%	12.50%	0.00%	21.05%
Phocaeicola	0.00%	19.61%	0.00%	0.00%	12.50%	23.08%	29.41%	14.71%	0.00%	12.50%	0.00%	25.93%	12.50%	12.50%	50.00%	15.79%
Porphyromonas	50.00%	25.49%	50.00%	50.00%	16.67%	34.62%	17.65%	29.41%	50.00%	16.67%	50.00%	33.33%	0.00%	25.00%	25.00%	36.84%
Prevotella	51.92%	21.57%	33.33%	67.86%	12.50%	30.77%	11.76%	26.47%	33.33%	12.50%	67.86%	29.63%	0.00%	18.75%	25.00%	31.58%
Pseudomonas	7.69%	0.00%	8.33%	7.14%	0.00%	0.00%	0.00%	0.00%	8.33%	0.00%	7.14%	0.00%	0.00%	0.00%	0.00%	0.00%
Rothia	82.69%	1.96%	87.50%	82.14%	0.00%	3.85%	0.00%	2.94%	87.50%	0.00%	82.14%	3.70%	0.00%	0.00%	0.00%	5.26%
Schaalia	69.23%	5.88%	62.50%	75.00%	0.00%	11.54%	5.88%	5.88%	62.50%	0.00%	75.00%	11.11%	0.00%	0.00%	12.50%	10.53%
Selenomonas	17.31%	54.90%	25.00%	10.71%	54.17%	53.85%	52.94%	55.88%	25.00%	54.17%	10.71%	55.56%	62.50%	50.00%	50.00%	57.89%
Solobacterium	36.54%	1.96%	41.67%	32.14%	0.00%	0.00%	5.88%	0.00%	41.67%	0.00%	32.14%	3.70%	0.00%	0.00%	12.50%	0.00%
Staphylococcus	75.00%	43.14%	79.17%	71.43%	50.00%	38.46%	35.29%	47.06%	79.17%	50.00%	71.43%	37.04%	37.50%	56.25%	25.00%	42.11%
Streptococcus	98.08%	86.27%	100.00%	100.00%	83.33%	88.46%	88.24%	85.29%	100.00%	83.33%	100.00%	88.89%	87.50%	81.25%	87.50%	89.47%
Tannerella	0.00%	37.25%	0.00%	0.00%	37.50%	34.62%	35.29%	38.24%	0.00%	37.50%	0.00%	37.04%	37.50%	37.50%	37.50%	36.84%
Treponema	0.00%	35.29%	0.00%	0.00%	33.33%	34.62%	35.29%	35.29%	0.00%	33.33%	0.00%	37.04%	37.50%	31.25%	25.00%	42.11%
Veillonella	92.31%	29.41%	95.83%	89.29%	29.17%	26.92%	17.65%	35.29%	95.83%	29.17%	89.29%	29.63%	0.00%	43.75%	25.00%	31.58%
